# Sport mega-event fantasies to financialization: the case of Porto Maravilha

**DOI:** 10.3389/fspor.2023.1113845

**Published:** 2023-06-29

**Authors:** Zainab Bhimani, Amanda De Lisio

**Affiliations:** School of Kinesiology and Health Science, Faculty of Health, Toronto, Canada

**Keywords:** sport mega-event, financialization, neoliberal urbanism, Rio de Janeiro, Brazil, Porto Maravilha.

## Abstract

This paper examines the relationship between sport mega-event construction and the financialization of housing in Rio de Janeiro. It focuses on the area of Porto Maravilha, constructed prior to the 2016 Olympic Games, and the particular use of the 2001 federal Statute of City and 1995 Strategic Plan for Rio de Janeiro to create new possibilities for neoliberal-capitalist expansion, initially disguised as democratized access to land yet, in effect, further commoditized land into a form of fictitious capital. To do so, we follow the work of Brazilian architect and author, Raquel Rolnik, and her argument that the legal-institutional emphasis on wealth distribution in urban legislation, propagated at the time of the internationally recognized sport mega-event in Brazil, was not adequately harnessed and instead used to endorse real estate speculation and uneven development in the metropolitan area. The coordination and collaboration between state and nonstate entities in mega-event construction is typically associated with deepened socio-spatial inequities, the privatization of public resource material, and the in/direct displacement of low-income communities. We review pertinent literature to better understand the role of sport mega-event fantasies in the construction of Porto Maravilha—which we come to understand as a speculative logic lubricant for finance. We do this to call attention to future studies to be particularly attuned to financialization in mega-event cities.

## Introduction

The relationship between sport mega-events and financialization is relatively unexamined in mega-event studies, even by studies that interrogate the known displacement/dispossession of local communities within host cities (1). Geographer Manuel B. Aalbers (2) defines financialization as “the increasing dominance of financial actors, practices, measurements, and narratives, at various scales, resulting in a structural transformation of economies, firms (including financial institutions), states, and households” (p. 2). Sociologist Greta Krippner (3) argues that financial activities (i.e., capital transferred with the expectation of future interest, dividends, or capital gains) now supersede profits from manufacturing activities in the United States. She uses the case of Ford Motor Company and its focus on earnings achieved through the sale of car loans (a financial product), in lieu of the sale of actual cars, as an example. We understand financialization as a process galvanized in contemporary times—since roughly the 1970s—that operates in concert with globalization and neoliberalization, and greatly contributes to the consolidation of wealth and income within wealthier classes and to the subsequent rise of inequality worldwide (4). With respect to sport mega-events (notably the Summer Olympics), we are familiar with studies that provide an overview of the neoliberal experimentation afforded through games construction, the devastating impacts on host communities in terms of gentrification, and we remain particularly cautious about the processes that now, in addition to displacing local communities, mobilize housing into an immaterial, tradable asset for financial investment.

Perhaps the most infamous cases of financialization are linked to housing and the growing dominance of financial actors in the housing sector. Of extraordinary note is the 2008–9 global financial crisis and foreclosure crisis, which was catalyzed by the treatment of home loans into tradable securities for global investment. The fallout of the treatment of mortgages as assets for financial investment disproportionately impacted low-income and racialized homeowners in the United States and continues to pose significant challenges to ensuring the right to adequate housing, globally. As August (5) suggests, “Financial investment in housing—via mortgage-backed securities, direct ownership, indirect investment—is dominating the global economy, enriching elites engaged in the financial sector and driving crises of affordability and inequality for low-income, middle-income, and working people” (p. 18). Real estate comprises 60% of global assets (i.e., US$217 trillion in 2016), 75% of which is represented by residential real estate (6) and reached an estimated global value of US$327 trillion in 2020 (7). Former UN Special Rapporteur on Affordable Housing Leilani Farha (2014–20) defines the financialization of housing and its consequences as follows:

The financialization of housing refers to structural changes in housing and financial markets and global investment whereby housing is treated as a commodity, a means of accumulating wealth and often as security for financial instruments that are traded and sold on global markets. It refers to the way capital investment in housing increasingly disconnects housing from its social function of providing a place to live in security and dignity and hence undermines the realization of housing as a human right. It refers to the way housing and financial markets are oblivious to people and communities, and the role housing plays in their well-being. (8, p. 3)

Following this definition, studies on the financialization of housing tend to overwhelmingly emphasize its consequences, without significant attention afforded to the essence and internal workings. As Christophers (9, p. 232; emphasis in original) writes, “Financialization is a politically limited critique insofar as it is essentially a critique of what finance does, especially elsewhere—of where its tentacles extend to, of the constituencies thus enrolled and ensnared, of the ‘nonfinancial’ logics thus adulterated—and not of what finance *is*.” The development of Porto Maravilha, launched in view of the 2016 Summer Olympics, is a useful case to examine not only for studies on the financialization of housing but also with respect to the literature on sport mega-events and human rights (10–12).

At the time of the 2014 FIFA World Cup, Porto Maravilha remained a largely speculative project, mostly removed from tourist traffic, yet, prior to the 2016 Summer Olympics, it was entirely transformed with new museums (one designed by Spanish “starchitect” Santiago Calatrava), an aquarium, light-rail vehicle system (VLT [Veículo Leve sobre Trilhos]), and commissioned artwork that celebrated diversity and signaled to the historic relevance of Afro-Brazilian and Indigenous cultures. The project comprised five million square meters of mainly public land and zoned an area of special urban interest (i.e., Zonas Especiais de Interesse Social, ZEIS, or Special Zones of Social Interests in English) one month after the successful 2016 Olympic-bid announcement. Local authorities—alone and in partnership with the private sector—have used large-scale urban operations to establish planning and policy procedures that are less democratic and heighten socioeconomic polarization (13). In Brazil, the role of the private sector is often presented as a “magic formula” that makes mega-urban projects feasible in times of fiscal crisis, without an excessive dependence on the public treasury (14).

This literature review demonstrates the ways that Porto Maravilha harnessed the momentum of the Olympic Games and the associated legislative reforms, which initially or allegedly were designed to promote the social function of land and recognize the historic struggle for certain housing rights, in order to bolster the real estate state (15). Instead of securing rights to occupy land, recent legislative reforms in Brazil were co-opted by finance to manufacture the “rights” to additional construction potential (i.e., Certificado de Potencial Adicional de Construção [CEPAC; Certificate for Potential Additional Construction]). As Former UN Special Rapporteur on Affordable Housing Raquel Rolnik (2008–14) said an interview, “After years of political struggle for the recognition of certain rights for those who have occupied land irregularly, it is as if those rights didn't mean anything any longer . . . the only thing that matters [for the city] is to put in movement a machine for the production of cities, internationalized and financialized” (qtd. in (16), para. 35). The growing dominance of financial actors, practices, et cetera, in the housing sector is a global trend (see also (17)), and thus it, unsurprisingly, informed legacy projects affiliated with the Olympic Games in Rio de Janeiro—especially given the history of the port region. We offer a brief overview of the history of development in the port region before we describe its contemporary refashioning as Porto Maravilha.

## *Bota abaixo* Accumulation in the Port District

The port region was historically integral to, and shaped by, the insertion of Brazil into the global economy—through the transatlantic slave trade, industrialization, and Eurocentric modernization. As Costa et al. (18) indicate, “This piece of earth was irreversibly integrated into the (pre)history of modernity and capitalism” (p. 4). From sugar and slave trade to gold and coffee, four centuries of extraction inform and impact realities and define social categories and hierarchies throughout Rio de Janeiro—and Brazil, more broadly. For more than three centuries (mid-sixteenth century until the mid-nineteenth century), slavery was the heart of the Brazilian economy. Brazil received approximately 4.9 million enslaved Africans through the transatlantic slave trade, representing roughly 40% of the 10 million enslaved Africans brought to the Americas (19). Archaeological studies estimate that roughly 500,000 to 1 million enslaved Africans entered Brazil via the wharf known as Cais do Valongo [Valongo Wharf], established in 1811. The Valongo Wharf in the port region of Rio de Janeiro operated as a pinnacle site for various activities related to the slave trade, until it was buried in 1843 in preparation for the arrival of Princess Teresa de Bourbon from Europe and renamed Empress Wharf. With the end of slave traffic (1850) and the abolishment of slavery (1888), formerly enslaved Africans (approximately 10,633 from Bahia; see also (20): 264) migrated to the port region of Rio de Janeiro and contributed to the development of Little Africa, a critical center for Afro-Brazilian cultural expression and resistance against social and racial oppression.

Soon after, Rio de Janeiro became the capital of the Republic of Brazil in 1889, and the port region was designated a federal district in 1891. With the election of Mayor Francisco Pereira Passos (1902–6) and financial support from President Rodrigues Alves (1902–6), an ambitious development plan, inspired by the Haussmann renewal of Paris (1853–70), was initiated. Roughly twenty thousand people (21, p. 62) were removed, and African music, dance, and religious ceremonies were criminalized. Construction typical of autoconstructed communities was also regulated (22, 23). Decree 391 (10 February 1903), for example, established new requirements for construction approval, including renovations and repairs to buildings and façades, and stipulated which materials were allowed (19). Furthermore, professional builders and building and floor–plan permits became mandatory (24). These regulations proved particularly harmful—especially within a region that notoriously housed newly freed Africans and immigrants, as well as residents dependent on the irregular occupation of land and its precarious title—and helped establish the first favela in Brazil, Morro da Providência.

Patterns of racial segregation were further exacerbated as local authorities prepared for the arrival of King Albert and Queen Elizabeth of Belgium in September 1920. The people of the Castelo neighborhood were especially threatened with eviction and demolition of their homes. Large-scale development prioritized capacities for major cruise-line traffic, which further integrated Brazil within global markets through a common point of arrival and departure for tourists (18, p. 65). Leu (25) documents urban renewals and rebellions in the period prior to Latin America's first world fair, hosted in Rio de Janeiro from 7 September 1922 to 23 March 1923: “The demolition frenzy [in the port region] constituted a violent attack on the city and its humblest inhabitants, making way for the construction of elegant avenues, monuments, and high cultural institutions” (p. 37). Caulfield (26) notes the rise of the phrase “for King Alberto to see” in popular presses at the time, as an idiom later adapted to “for the English to see,” meaning “to do something merely for show” (p. 64). Forced evictions and displacements were articulated through the supposedly apolitical discourse of hygienization (27), which resourced fear of infectious disease (such as venereal disease, smallpox, yellow fever, and tuberculosis) yet especially targeted Afrodescendant and immigrant communities (28). Large-scale urban development served to reinforce racial hierarchies of power and governance, thus further integrating Brazil into global circuits of racial capitalism.

The supposed end of *bota abaixo* (knock-it-down) reforms, typically marked by the end of the Sampaio administration (1920–22), coincided with the rise of industrialization. Yet, with the 1929 stock market crash in New York, industrialization failed to become economically significant and instead remained subordinate to agricultural export industries. Rio de Janeiro, the initial industrial hub for Brazil, declined as a significant center, as activities shifted to urban peripheries. Through its decline, the port and its shipping infrastructure were unable to meet the new volume of both import and export industries, and the model of urbanity prioritized instead the long-demure quest for modernization (18). This resulted in the treatment of the port region as a mere transit route for travel from the newly constructed international airport and the middle-class Centro/downtown to the more affluent south zone/beachfront of Rio de Janeiro, namely, Copacabana and Ipanema.

In 1944, with the construction of Avenida President Vargas, during his dictatorship (1937–45), important cultural sites and neighborhoods predominately occupied by Afro-Brazilians were once again targeted, notably Praça Onze, a site of important cultural production, located in the center of the historic Cidade Nova neighborhood. Development decoupled these sites and neighborhoods from Centro/downtown through the construction of a sixteen-lane expressway. It also coincided with construction for the first FIFA World Cup in Brazil, hosted in 1950, from 24 June to 16 July. This was the fourth World Cup ever hosted, after the tournament was canceled in 1942 and 1946 due to World War II. With the World Cup, Brazil debuted Maracanã Stadium (29), which was located roughly seven kilometers slightly north of the port region. After the World Cup, a new international airport was unveiled in 1952. Traffic from the airport could easily pass through Centro/downtown and the adjacent port region, directly to Copacabana and Ipanema, through Avenida Presidente Vargas and the Perimetral Bridge. Urban development followed this route and was concentrated along the beachfront, rendering Centro/downtown and the adjacent port region as less and less significant, particularly with the movement of federal capital from Rio de Janeiro to Brasília in 1960.

To our current knowledge, there is limited literature on urban development in Centro/downtown and the port region amid the military dictatorship. This period is also referred to as the Fifth Brazilian Republic, which was established on 1 April 1964-after a coup d'état by the Brazilian Armed Forces, with support from the United States government, against President João Goulart–and ended on 15 March 1985. Following the protracted transition from military rule, elected authorities in Rio de Janeiro focused on the Olympic Games as an avenue to coordinate massive investment and revitalization, following Barcelona and the 1992 Summer Olympic Games. Vainer (30–32) and de Oliveira Leal et al. (33) attribute the administration of Mayor Cesar Maia (1993–96; 2001–9) as a significant driver of mega-event fantasies and, specifically, the decision of municipal authorities to hire a consortium of Catalonia companies in 1993 to prepare the bid to host the 2004 Summer Olympic Games. The bid was ultimately crafted and proposed an urban model inspired by Catalonia's capital, Barcelona, which focused on the waterfront as an important site of renewal.

Unsuccessful at the time, the process solidified a partnership between city hall and private entities which acted in consultation with Tecnologies Urbanes SA (34), and resulted in the creation of urban strategies (articulated within the 1995 Strategic Plan) that specifically sought to reimagine and reinvent the port region of Rio de Janeiro based on the Olympic redevelopment of the port in Barcelona (33, 34).^1^ The model pursued in Barcelona—by which the 1992 Olympic Games produced major opportunities for the commodification of culture and accelerated processes of urban revitalization that relied upon cooperation between the public administration and private sector to develop tourism—led to the liberalization of land, increased presence of international real estate investment, and the eventual expulsion of existent communities.

Profit potential through “monopoly rent” (35) and the prioritized integration of Barcelona into global economies created a new export, the “Barcelona model” for urban reform, which was adopted by Olympic-hopeful cities beyond Brazil.^2^ Monopoly rent, typically characteristic of the accumulation strategies pursued in Barcelona in the wake of the Olympic hype, was dependent upon wealth extracted from a material “special quality resource” (35, p. 94) that increased in value through the commodification of culture. Wealth, in this case, was captured by a small powerful segment of the local bourgeoisie to whom Juan Antonio Samaranch, the president of the International Olympic Committee, was integral. This is a marked difference from the finance-led accumulation strategies observed more recently in the construction of Porto Maravilha, which depended not on a material “special quality resource” but rather on the purchasable “right” to future construction potential.

Amid the pursuit of a sport mega-event in Rio de Janeiro and consultation with Catalonian expertise in the creation of an Olympic bid, Brazil suffered a significant debt crisis, which resulted in the contentious introduction of fiscal austerity and monetary policies.^3^ Domestic companies started to favor a finance-led regime of accumulation, which triggered “appreciating fictitious capital to the detriment of productive capital, with the exacerbated profits of the financial system; increasing also private, but mainly public debt; increasing the wealth of the rentier segment of society” (36, p. 282, qtd. in (18), p. 60). The response to the debt crisis further cemented the role of finance within the national economy (37: 133). In the next section, we examine Porto Maravilha as a development project that is described as and still dependent on *bota abaixo* but also (and with growing dominance) tradable financial products or new asset classes.

## Porto Maravilha: Finance-led regime of accumulation

In the case of Porto Maravilha, the financialization of housing was assisted by a market-based land-value capture tool (known as a CEPAC), implemented through an agreement (known as an Urban Partnership Operation [UPO]) and funded through the national worker-indemnity fund. The monetization of additional construction potential is unique; the financialization of housing typically involves the conversion of mortgages, houses, apartments, mobile homes, et cetera, into financial products. But CEPACs, property rights for the development of airspace beyond the legislated limit, signify a relatively unexamined empirical terrain for research into urban financialization. Since the financialization of housing is a subject of heightened scholarly attention (38), an overview is provided—with particular attention to the ways this literature intersects with studies on the sport mega-event.

Sport mega-event studies overwhelmingly analyze the sport mega-event as a mechanism (or manufactured crisis) through which processes, policies, and personalities coalesce to realize various strategies of neoliberal urbanism. The term neoliberal urbanism (44–47) is commonly used to refer to “urban specificities of a macroeconomic process, in which the dominant accumulation pattern promotes a ‘market-oriented regulatory restructuring’ (48), in unequal and heterogeneous social, political, and institutional settings” (49, p. 77), with competitiveness as innate and the contribution to “advanced marginality” (50) as inevitable (51–55). With common trajectories in the Global South and North, neoliberal urbanism is variegated, path dependent, uneven, and critically understood as a “moving matrix of articulations, predicated on conditions of existence that necessarily involve programmatic incompleteness and contradictory cohabitation both in—and, once again, across—multiple sites, struggles, situations, and settings” (56: 247).

The nexus between financialization or a finance-led regime of accumulation (as manifest and expressed through the creation of a fictitious economic circuit) and neoliberal urbanism is complicated and conflicted. Within urban studies, it is argued that financialization is intrinsic to neoliberalization (57), entrepreneurial strategies are increasingly realized through financialization (58), and financialized urbanism is not a new phase of neoliberal urbanism but the avenue through which the latter was enabled (59). Neoliberalism is an agenda through which a more favorable regulatory and ideological terrain can be established for financialization, even as one can also locate particularities in which the implementation of neoliberal urbanism is clearly on course and financialization is constrained. Thus, financialization is not necessarily an outcome of neoliberalism, as argued by Pereira (60), but a process that emerged alongside neoliberalism, and it is likely to propel and be propelled by similar policies, processes, and personalities.

There are at least two realities in Brazil that complicate studies on the sport mega-event, neoliberal urbanism, and the displacement of marginalized communities, and are relevant to our focus on the relation between the sport mega-event and the financialization of housing in host cities. First, formalized access to housing—whether provided by the state or private sector—has never been universal in Brazil, as is typically imagined to be the case in the Global North (61, 62). Furthermore, studies that describe a (dismantled) welfare state, deregulation, and/or privatization of social services or human rights—often associated with neoliberal urbanism in Anglo-American literature—are also inaccurate regarding the Brazilian case. Instead, analyses based in Brazil, and Latin America more broadly, describe a specialized and professional real estate development sector, comprised predominately of construction companies but also inclusive of insurance and finance. For example, in 1964, the military government of Brazil launched a public bank specializing in housing finance: the Banco Nacional de Habitação (BNH; National Housing Bank), which existed until 1986. The BNH was funded primarily through a worker-indemnity fund (Fundo de Garantia por Tempo de Serviço [FGTS; Length-of-Service Guarantee Fund], described in the next section) created in 1966, which is still in existence, was used in the development of Porto Maravilha, and is funded through a percentage of worker salaries deposited into a private account that is publicly managed (39). Since 1970, this fund has diversified from the residential market to now financing urban infrastructure, yet it is still a major financer of home ownership and contributor to the Brazilian housing market.

Second, housing in Brazil is not typically approached as a low-risk investment, due to the limited availability of credit, high cost of credit, and the structural reliance on volatile external investment (63). Maricato (64) surmises, “There is a formal or legal capitalist market for a small portion of the population, a luxury market that is highly speculative” (p. 23). That said, the Brazilian mortgage market has rapidly developed and is now one of the largest in Latin America, a trend that is often equated to the growth of the financial sector and improved access to credit via, for example, the federal program, Minha Casa, Minha Vida (My House, My Life) (65). In this environment, financialization emerged alongside policies conducive to neoliberalism: first, by opening a regulatory and ideological terrain, initially disguised as democratized access to land that, second, rendered land and real estate to calculative processes of abstraction.

Through his analysis of financialization in Brazil, Pereira (60) defined financialization as distinct from neoliberalization, as “the expression of an increasing influence of financial rationales, narratives and calculative practices in different spheres of life” (p. 604), and explained that “the rise of these calculative practices is rooted in a continued process of value abstraction, in which property rights over commodities that are concrete bearers of value are increasingly represented by fictitious assets, giving rise to an autonomous economic sphere where ideal claims upon value are exchanged” (p. 606). In the case of Porto Maravilha, the right to additional construction potential was sold, purchased, and financed through a national worker-indemnity fund, which subsequently tied the worker to real estate valorization in a city of (already) immense unaffordability. This was facilitated through legislation, which was important in establishing Porto Maravilha as the largest public-private partnership in Brazilian history.

After the successful Olympic-bid announcement in October 2009, municipal authorities established Porto Maravilha as a special urban-interest zone (via the newly enacted Complimentary Law 101/2009) and created a public-private partnership between the municipal government and the Porto Novo Consortium, comprised of three of the largest construction and engineering companies in Brazil: OAS Ltd., Norberto Odebrecht Brasil, and Carioca Christiani-Nielsen Engenharia (via the newly enacted Complimentary Law 102/2009). This created the UPO referred to in English as the Urban Development Company of the Port Region of Rio de Janeiro (Companhia de Desenvolvimento Urbano da Região do Porto do Rio de Janeiro [CDURP]). The use of a UPO, officially legislated within the 2001 Federal Statute of the City, was initially inspired by the *solo criado* (created land) philosophy, or the idea that property improvement should not be reserved or appropriated solely by the property owner. According to Abigail Friendly and Ana Paula Pimentel Walker (2022), “Solo criado was enacted through a tool known as *outorga onerosa do direito de construer* (OODC), charging developers for additional building rights by exchanging urban improvements of social interest to the community, making OODC—theoretically—a redistributive tool” (pp. 1172–73).

Several studies have since argued that the policy instrument of a UPO, although initially intended as a redistributive tool, is increasingly harnessed as “political devices that help facilitate the entanglements between the state and viewpoints, calculative practices, and valuation techniques that are specific to financial markets” (66, para. 1) and thereby more accurately understood as a “financializing policy instrument” (67). Furthermore, Klink and Stroher (68) argue that the specific Porto Maravilha UPO marked a notable shift in UPO governance in Brazil, which historically relied upon a tripartite deliberative council with participation from the real estate sector, academia, civil society (most notably in the form of community and social-movement representation), and local government; the Porto Maravilha UPO subsequently eroded potential for civic participation, as it limited the capacity for local governments to monitor and evaluate the activities of companies (specifically their executive, management team, etc.). This public-private partnership allowed the UPO to move forward without democratic oversight, thus streamlining its management and implementation without the need for elected officials or local communities to be represented in development decisions related to the project.

Interestingly, the use of a UPO (such as CDURP) was only legalized through the 2001 Federal Statute of the City, which required all major cities in Brazil to facilitate new participatory processes and establish a new development plan based on these participatory processes by 2006—the same year the 1995 Strategic Plan was set to expire. Some argue that the CDURP was established in 2009 in a legal vacuum between the expiration of the 1995 Strategic Plan (in 2006) and the ratification of the new plan in 2011 (69). As an urban consortium/partnership, CDURP was afforded flexibility (i.e., through a simplified regulatory framework, streamlined permit process, and tax incentive) to act without democratic oversight in exchange for the provision of activities formerly assigned to public agencies (e.g., landscape maintenance, garbage collection and removal, rainwater drainage, public illumination, restoration, reuse of properties, etc.) for a fifteen-year period from 2009 to 2026. Furthermore, in lieu of the Municipal Housing Secretary (Secretaria Municipal de Habitação [SMH]), CDURP was legally entitled to evict people who interfered with project plans.

In October 2009, the municipality announced the removal of more than 3,500 families to make way for the development of Porto Maravilha (39: 252). By May 2011, 800 families in Morro da Providência were reportedly threatened with removal. By November 2013, 430 families were evicted, with an estimated 196 families specifically from Morro da Providência (70, 71). Many families were never adequately compensated (i.e., they were compensated for their home but not for the physical land), nor did they receive decent resettlement options. Local authorities rationalized the clearance of entire communities as necessary due to high-risk dangers, such as environmental hazards or structural/engineering insecurities, or simply because the land was needed for mega-event construction (39: 252). With the transfer of wealth (in the form of public land) and the erosion of democratic oversight (through the establishment of the largest private-public partnership in Brazil), the area was “marked by the accelerated demise of a planning vision in the service of the public interest, to be replaced by a competitive, entrepreneurial, and economistic conceptualization of the city; characterized by privatization and the search for revenue generation” (72, p. 148). In total, the transfer represented five million square meters of mainly public land, which already housed roughly 40,000 people. The federal government also contributed 0.45% of the R$24 billion spent on Olympic legacies to the project.

## From finance-led to fictitious capital in Porto Maravilha

The policy instrument of a UPO (i.e., CDURP), funded through the sale of additional construction potential (CEPAC), was initially intended as a redistributive tool (i.e., articulated via the *solo criado* philosophy) yet was instead co-opted by finance: a process streamlined through the “commodification of culture” (35) afforded through the globally recognized sport mega-event. From the outset, Porto Maravilha transferred public land to private companies, rationalized via imaginaries of a mixed-use, high-end neighborhood with luxury residential properties, commercial businesses, and cultural facilities and amenities in a supposedly “abandoned” area. By 2011, Porto Maravilha experienced intense financial appreciation. The Olympic Boulevard, combined with the festivities set to occur in the area, heightened the hype [e.g., Coca Cola Parade, Samsung Galaxy Studio, a bungee jump sponsored by Nissan, the exhibition *Se Prepara Brasil* (Get ready, Brazil), the Skol Panoramic Ballroom and Brewery, and Nike Shop]. Mayor Eduardo Paes stressed the importance of Porto Maravilha to the Olympic Games, and the *Associação de Dirigentes de Empresas do Mercado Imobiliário* (ADEMIRJ; Association of Real Estate Management, Rio de Janeiro) estimated an increase in the area price per square meter by 300%. The cost of construction was estimated at R$8 billion. To finance the project, the Porto Maravilha UPO relied on the sale of additional construction potential, known as CEPAC, a municipally issued bond or certificate, which guaranteed the owner the right to airspace beyond the legally stipulated height limit of four stories. The financial mechanism of a UPO is described in the work of Mosciaro et al. (73, p. 2164) as follow:

The establishment of relatively flexible zoning rules inside the perimeter of the UO [Urban Partnership Operation] is one of the key elements of this framework’s financial engineering. The mechanism works as follows: the zoning rules applicable inside the perimeter of the UO define standard and maximum floor area ration (FAR). After buying the land a developer can carry out, free of further charge, a project in which the total built area does not exceed the standard FAR. Projects that exceed this ratio are also admissible if the developer purchases additional development rights. Nevertheless, such additional development rights cannot exceed the maximum FAR limit. The acquisition of additional development rights is effected through the purchase of financial titles issued by the municipal government, called CEPACs. An UO’s law defines the total amount of CEPACs that can be issued during its implementation. It also sets different parameters for the conversion of CEPACs into development rights, regulating their spatial distribution within each intervention.

The use of a CEPAC to fund a UPO was initially adopted in São Paulo in 1995, before the Statute of the City officially legislated the use in 2001. Initially, revenue collected from the sale of each CEPAC would be reinvested within the perimeter of the UPO to fund activities that were previously publicly subsidized (e.g., road construction and improvement, waste management and collection, public transportation, social housing, etc.). However, the sale of a CEPAC also evolved into an avenue for a buyer to be articulated into the land and property market, which thereby made a CEPAC a market-based value-capture tool that could be traded or used as credit money. In the case of Porto Maravilha, additional construction potential was detached from any concrete real estate project and is thus often described as a form of fictitious capital issued by municipalities (73, 75). The right to additional construction potential is a financial asset with autonomous value, detached from concrete real estate; the mere “right” to build becomes a financial product.

In June 2011, the Caixa Econômica Bank (Caixa), a federally owned financial institution, became the monopolistic buyer of every available CEPAC (6.4 million) in the Porto Maravilha UPO for R$3.5 billion. These were to be resold and negotiated on the market with, for example, construction companies, which would in turn receive the right to build in the area. To finance this purchase, Caixa borrowed from the FGTS, a mix between a pension fund and unemployment insurance, to create a Real Estate Investment Trust (REIT Porto Maravilha). The FGTS is a mandatory deposit of 8% deducted from employee salaries by their formal employer in Brazil. A worker can access this fund to finance home ownership, which has made home ownership more accessible in the nation. However, the decision to use this fund in recent large-scale urban reform has increasingly tied the worker fund to the valorization of land and associated real estate. As a federally owned financial institution, then, the bank (and by extension, the state) is forced to perform as an active agent in gentrification (73). It is estimated that a square meter of residential or office space will need to sell for at least R$10,000 (approximately US$2,800) for the investment to be profitable—and thereby house some of the most expensive real estate in Rio de Janeiro, a metropolis with real estate and rental properties that already far exceed most global cities in market value (72). If families and small commercial activities were not directly evicted in construction, real estate speculation and private investment almost immediately made the neighborhood too expensive to afford.

Finance-led accumulation strategies are distinct from *bota abaixo* accumulation, yet they still involve the displacement of low-income communities. Between 2009 and 2015, 22,059 families were removed from Rio de Janeiro for Olympic construction, or an estimated 77,206 people (39: 20). Broudehoux (43) notes that “a great proportion of revanchist policies put in place in the context of mega-event preparation overwhelmingly affected the Afro-Brazilian population and their cultural practices” (p. 142). Faced with an anticipated population increase of 70,000 people by 2025 in the port region, Adriana, a long-time resident of Morro do Pinto, a favela in the region, explains, “There used to be an affordable supermarket in Santo Cristo, but with the construction, it closed. The [Porto Maravilha] project required the property. All the real estate has increased in value and so too did the price of everything else. So, I need public transit to travel to Paça da Bandeira [North Zone] for groceries and basic supplies. It is such a hassle, especially without a car” (Personal communication conducted in Portuguese, 16 January 2017).

Redevelopment failed to include a detailed plan to allow communities historically housed in the area to remain—of which 94% of the families earned less than R$2,640 in 2015 (73). Paes celebrated the project in a supposedly abandoned region of Rio de Janeiro: “What we did in the Porto Region was practically rebuild, revamp a consolidated area of the city that had been abandoned, realizing what is most important: that is, people starting to live in the Porto Region” (74; our translation). Funded through the FGTS, Porto Maravilha paradoxically worked against the immediate interest of the worker. As Mosciaro et al. (73) describe,

The practices adopted by FGTS in this project also give clear signals of its financially driven motivations. As a fund that was created to guarantee welfare provision and enhance savings of workers, one could argue that a profitable implementation of the Porto Maravilha project would ultimately result in higher returns for employees benefiting from the fund. However, to achieve these profits, FGTS, along with other (semi-)public authorities is undertaking actions that go against the class interests of workers. Pension funds are typically prone to cope with pressures to push wages down to enhance profits of the companies in which they won stocks. In a similar way, the FGTS displaces low-income households for the sake of higher profits (pp. 16–17).

While autoconstructed and insecurely tenured and titled communities were beyond the CEPAC perimeter, reconstruction still insisted on the removal of nearly half of the families from the Providência hillside, and more than one thousand families that squatted in the asphalt or flatter areas of the region were evicted in the first year of the project.^4^ In terms of the class interests of workers, the model knowingly places people in a neoliberal strait jacket by gambling their future savings on the valorization of an already unaffordable housing market.

Since 2014, Porto Maravilha UPO has remained in financial distress. The extended economic and political crisis in Brazil shattered the expectation of profit within the area. In 2015, 90% of all the issued CEPACs remained unsold, as it was reported that the construction company Carioca Engenharia allegedly bribed Eduardo Cunha, then president of the Chamber of Deputies, with R$52 million (US$13 million) to use his influence to help Carioca Engenharia obtain the R$3.5 billion from FGTS. All three construction companies that comprise the Porto Novo Consortium have now been indicted, with some executives even sentenced and imprisoned.^5^ In 2016, a social housing plan was proposed for Porto Maravilha, which vaguely called for the construction of five thousand residential units—nearly a decade after the inauguration of the UPO and after the eviction of precariously tenured families from the area. This plan has not yet and is unlikely to ever materialize. The land designated for social housing development—Praia Formosa, Usina do Asfalto, and Pátio da Marítima—was sold to guarantee the transfer of R$1 billion to Porto Maravilha UPO (39). Between July and November 2017, there was a partial suspension of activities in the area due to the failure of the government to pay the Porto Novo Consortium. By 2018, investor interest hit rock bottom. While Porto Maravilha is emblematic of the way in which land is transformed in order to accrue capital—more recently through monetized speculation, albeit an unfinished project—and financed entirely through public investment, it is hard to imagine a result that is favorable to the local populace. On the one hand, the sale of the CEPAC for profit implies the further removal of low-income, predominately Afro-Brazilian communities from the area. On the other, if the desire for additional construction potential in the area is further squandered, it would entail massive financial losses to FGTS, the national worker-indemnity fund.

## Conclusion

In the 1988 Constitution, an urban agenda emphasized (1) the social function of cities and properties; (2) the right to the city, particularly for people in autoconstructed communities with insecure, precarious title and tenure; and (3) participatory development processes that incorporated citizen engagement (39, p. 207). In total disregard of the Constitution, urban processes which followed the sport mega-event used the spectacle to drive a speculative logic. This benefited the financialization of housing through the purchasable and tradable “right” to additional construction—a new and arguably more aggressive complement to neoliberal urbanism, in that land and the associated housing and real estate are detached from their material form and inserted into a fictitious economic circuit. Financialization is driven by the financial interest of an investor and/or financial institution. Ironically, in the case of Porto Maravilha, a worker-indemnity fund is the primary investor and thereby dependent on the valorization of real estate—even as valorization further prices the average worker (and future generations) out the housing market. Mosciaro et al. (73) argue, land and the right to additional development are treated literally as a financial asset that can be purchased and exchanged, indicative of a shift from entrepreneurial to more finance-led accumulation strategies.

Rolnik (39, 77) is especially relevant to urban studies on neoliberal urbanism and financialization but often overlooked in sport mega-event literature, even as the event and subsequent analysis are increasingly located in the Global South, so-called peripheral economies. Throughout our investigation, we remained particularly inspired by her effort to illustrate that, despite the discursive focus on wealth distribution in urban legislation, propagated with other more modern and progressive fantasies of the sport mega-event, the long-fought rights-based approach to housing in Brazil must now contend with finance. As host of the 2007 Pan/Parapan American Games, 2010 World Urban Forum, 2011 Military World Games, 2013 World Youth Day, 2014 FIFA World Cup, and 2016 Olympic Games, Mayor Paes made bold claims that event investments would extend to all 582 favela communities in Rio de Janeiro through intentional integration. His projections reinforced the pledge of President Luiz Inácio Lula da Silva, who equally promised to use the Olympic Games to urbanize favelas in Rio de Janeiro. We attempted to follow the urban scholarship inspired by Rolnik (39, 77) in order to trace the ways in which urban legislation in mega-event Rio de Janeiro (devised with an emphasis on wealth distribution) intervened and coalesced to not only gentrify host communities but also increase the influence of finance. Ultimately, we can expect to see the same or similar processes in future host cities as finance plays a growing dominance in the housing sector—through mortgage-backed securities, securitization of housing debt and loans, tax increment financing (in the United States or Canada), airspace rights, or CEPACs (in Brazil).

## Footnotes

^1^The Strategic Plan adopted a mode of urban governance developed in consultation with experts from Barcelona, who were approached for their acclaimed Olympic revitalization, which required large-scale urban reform and celebrated the mega-event as a viable method to bolster tourism, stimulate inward investment, and ultimately improve the global image of Rio de Janeiro (40). As Gaffney (41) notes, “The 1995 Strategic Plan laid out a framework for urban governance that would make the city ‘competitive’ by employing the strategies of city-marketing, the top-down implementation of large urban renewal projects, and the pursuit of a political economy based in urban entrepreneurship” (p. 219).

^2^In Toronto, Canada, for example, the failed 2008 Olympic bid facilitated a trilevel policy coordination among the City of Toronto, province of Ontario, and the federal government, each of which promised C$500 million to waterfront development, in order to establish Waterfront Toronto, “a publicly funded corporation that is significantly influenced by finance capital and real estate development interests, but relies on a commitment from the provincial government in both planning and policy” (42, p. 679).

^3^In 1994, the Plano Real was launched as a method of controlling inflation; it introduced a new currency and implemented legislative reform that aimed to modernize the current banking system in Brazil and facilitate a larger influx of international capital (39, p. 212). Real estate was an important target of financial capital—for example, Certificado de Recebíveis Imobiliários (Mortgage—Backed Securities) and Fundos de Investimento Imobiliário (Real Estate Investment Trusts [REITs]) were introduced, as well as new regulations for mortgage—securitization companies—but did not yet signal a process of financialization.

^4^Faulhaber et al. (76) demonstrated that families received on average R$28,000 between 2009 and 2012 for the compensation of building material, and most families who were evicted did not have property title and were not compensated for land or the labour required in the construction of their house; the maximum indemnity amount, established by municipal decree, is R$62,000.

^5^The poor performance of CEPAC secondary market may be regarded as a limit to financialization; yet, as Mosciaro et al. (73) argue, “their issuance still represents the transformation of development rights into a form of fictitious capital, making room for the intensification of speculative logic” (p. 2174). To this speculative logic, we add, the sport mega-event was critical.
